# Associations of non-high density lipoprotein cholesterol and traditional blood lipid profiles with hyperuricemia among middle-aged and elderly Chinese people: a community-based cross-sectional study

**DOI:** 10.1186/1476-511X-13-117

**Published:** 2014-07-23

**Authors:** Juan Xu, Hao Peng, Qinghua Ma, Xiaohua Zhou, Wenxin Xu, Liang Huang, Jiarong Hu, Yonghong Zhang

**Affiliations:** 1 Department of Epidemiology, School of Public Health, Medical College of Soochow, 199 Ren-ai Road, Industrial Park District, Suzhou 215123, China; 2 The Third People’s Hospital of Xiangcheng District, Suzhou, China

**Keywords:** Hyperuricemia, Blood lipids, Non-high density lipoprotein cholesterol

## Abstract

**Background:**

Increased serum uric acid (SUA) is associated with dyslipidemia. However, there are conflicting data about the role of single lipid species including non-high density lipoprotein cholesterol (non-HDL-C) in promoting SUA accumulation. Here, we aimed to compare non-HDL-C with other traditional blood lipid profiles in relation to hyperuricemia in a middle-aged and elderly Chinese population.

**Methods:**

Data was collected from 9580 participants undergoing routine physical examinations in Xiangcheng district of Suzhou. SUA, total cholesterol (TC), triglyceride (TG), low density lipoprotein cholesterol (LDL-C), and high density lipoprotein cholesterol (HDL-C) were examined for all participants. Non-HDL-C was calculated by subtracting HDL-C from TC. The associations of blood lipid profiles with hyperuricemia were examined in men and women, respectively. The areas under Receiver Operating Characteristic (ROC) curves (AUCs) were compared to assess the discriminatory value of blood lipid parameters for predicting hyperuricemia.

**Results:**

All blood lipid parameters significantly correlated with SUA (all *P* values <0.001). The correlation coefficient between SUA and TG was the highest in both genders. The correlation coefficient of non-HDL-C was higher than HDL-C in males and was higher than TC and LDL-C but followed HDL-C in females. In male group, AUC of TG (0.659) was greater than that of non-HDL-C (0.595) (*P* values <0.001). The AUC values of HDL-C, TC and LDL-C were lower; respectively 0.581, 0.559 and 0.552. In female group, AUC was highest for TG (0.678) followed by HDL-C (0.616), non-HDL-C (0.610), LDL-C (0.559) and TC (0.557) (all *P* values < 0.001).

**Conclusions:**

In both genders, serum TG has the strongest association with hyperuricemia among blood lipid parameters. Non-HDL-C is also significantly associated with hyperuricemia.

## Introduction

Serum uric acid (SUA) is the final oxidation product of purine metabolism in the circulation. SUA may accumulate in the body due to increased production or decreased elimination [1]. High SUA is a prerequisite for gout [2] and is also associated with the metabolic syndrome (MetS) and its components [3] and consequently risk factors for hypertension, stroke and other cardiovascular diseases (CVD) [4-6]. Lipid profiles have been found to have a stronger association with SUA than other MetS components [3]. Though it is well established that SUA is closely associated with dyslipidemia, there are conflicting data about the role of single lipid species in promoting SUA accumulation [5,7-9]. A recent study in the Nigerians revealed that total cholesterol (TC) and low density lipoprotein cholesterol (LDL-C) were significantly associated with SUA [9], but such associations were not found in the Iranians [5]. Apparently, the relationship between lipids and SUA varied in different populations. In addition, a study has demonstrated that non-high density lipoprotein cholesterol (non-HDL-C) is more closely associated with CVD, compared with other lipid parameters [10]. However, there are no reports on relationship between non-HDL-C and SUA as well as comparison of the relationships between several lipid parameters and SUA level or hyperuricemia in Chinese population. In present study, we analyzed the associations of blood lipids including non-HDL-C with hyperuricemia, and compared the associations of the lipid profiles with hyperuricemia in a middle-aged and elderly Chinese population.

## Materials and methods

### Study participants

All residents aged over 45 years in ten communities located in Xiangcheng district in the suburb of Suzhou City were examined from June, 2012 to April, 2013. Totally, there were 9642 individuals who underwent a physical examination. The exclusion criteria were to meet one of the followings: (1) having clinical suspicion of chronic renal diseases or tumors; (2) history of chronic liver disease; and (3) self-reported gout. After the exclusions, 9580 participants (4246 male and 5334 female) were included in the final analysis. This study was approved by Soochow University Ethics Committee. All participants provided written informed consents.

### Data collection

All the examinations were performed by trained medical staff. Data on demographic, personal medical history were obtained using a standardized questionnaire. Standing height and body weight were measured with participants wearing light clothing and without shoes. Body mass index (BMI) was calculated as the ratio of weight in kilograms to height in meters squared. Three consecutive sitting blood pressure measurements (30 seconds between each) were taken by trained staff using a mercury sphygmomanometer according to a standard protocol, after the subjects had been resting for at least 5 minutes. The first and fifth Korotkoff sounds were recorded as systolic blood pressure (SBP) and diastolic blood pressure (DBP), respectively. The mean of the three records was used in analysis. All blood samples were obtained from the antecubital vein in the morning after a requested overnight fast (at least 8 hours). All laboratory tests were done immediately after sampling. All the blood samples were processed and analyzed using Roche Modular automatic biochemical analyzer (Roche Diagnostics Ltd, Mannheim, Germany). SUA was determined by the uricase method (Roche Diagnostics, Mannheim, Germany). We defined hyperuricemia as SUA > 416 μmol/L (7 mg/dL) for men and > 357 μmol/L (6 mg/dL) for women [11]. TC and triglyceride (TG) were measured by standard enzymatic colorimetric methods (CHOD-PAP and GPO-PAP; Roche Diagnostics, Mannheim, Germany). High density lipoprotein cholesterol (HDL-C) and LDL-C were measured by an enzymatic test (HDL-C-Plus and LDL-C-Plus; Roche Diagnostics, Mannheim, Germany). Non-HDL-C was calculated by subtracting HDL-C from TC. Fasting plasma glucose (FPG) was examined using a standardized reference technique (GOD-PAP method; Roche Diagnostics, Mannheim, Germany). The intra-coefficients of variation values were less than 5.0% and the inter-assay values were less than 9.5% for all examinations. In order to take knowledge of the liver and renal function of our study participants, some important biomarkers such as aspartate aminotransferase (AST), alanine aminotransferase (ALT), blood urea nitrogen (BUN), and serum creatinine (SCr) were also tested using commercial reagents.

### Statistical analysis

All statistical analyses were carried out separately by sex because of the varied level of SUA between genders [5]. Baseline characteristics of participants were presented and compared between participants with and without hyperuricemia. Comparisons in the means of continuous variables with a normal distribution were performed by using Student’s *t*-test. Comparisons in medians of continuous variables with a skewed distribution were performed by using a Wilcoxon rank-sum test. As blood lipids including TC, TG, LDL-C, HDL-C, and non-HDL-C and SUA values were not normally distributed, correlations between SUA and lipids were determined using Spearman’s rank correlation test. The associations of various blood lipids with hyperuricemia were evaluated using univariate and multivariate non-conditional logistic regression models. Odds ratio (OR) and 95% confidence interval (CI) for hyperuricemia were calculated. In the multivariate models, variables that differ between participants with and without hyperuricemia, including age, FPG, BMI, SBP, DBP, SCr, BUN, ALT, AST were included as covariates. Lastly, we assessed the relation of the five lipid indexes with hyperuricemia by computing Receiver Operating Characteristic (ROC) curves and comparing the areas under ROC curves (AUCs) with the Z-statistic [12]. All reported probability values (*P*-values) were based on two-sided tests and *P* value <0.05 was considered statistically significant. Statistical analysis was conducted using SAS statistical software (version 9.1, Cary, North Carolona, USA).

## Results

### Baseline characteristics

A total of 9580 participants (4246 men and 5334 women) with an average age of 60.9 years were included in the analysis. Among them, 1270 (13.26%) participants had hyperuricemia. The prevalence was significantly higher in males than that in females (16.23% vs 10.89%, *P* < 0.001). The baseline characteristics of participants with and without hyperuricemia in both genders are shown in Table 1. The participants with hyperuricemia tended to be older and have higher FPG, BMI, SBP, DBP, SCr, BUN, ALT and AST compared with those without hyperuricemia in both genders (all *P* < 0.05). Participants with hyperuricemia had higher serum TC, TG, LDL-C, non-HDL-C and lower HDL-C compared with those without hyperuricemia in male and female groups (all *P* < 0.05). There were no significant differences in heart rate between the two groups in either males or females.

**Table 1:** Baseline characteristics of participants by serum uric acid levels in genders

**Characteristics**	**Men**				**Women**	
	**≤ 416 μmol/L**	**> 416 μmol/L**	***P*-value**	**≤ 357 μmol/L**	**> 357 μmol/L**	***P*-value**
N	3557	689	-	4753	581	-
Age, years, mean ± SD	60.55 ± 9.78	62.10 ± 10.66	<0.001	60.34 ± 9.81	66.12 ± 10.74	<0.001
SBP, mmHg, mean ± SD	136.59 ± 18.86	140.18 ± 20.00	<0.001	140.46 ± 19.37	148.99 ± 19.75	<0.001
DBP, mmHg, mean ± SD	85.87 ± 11.61	87.98 ± 11.47	<0.001	83.74 ± 11.48	86.36±11.68	<0.001
BMI, kg/m^2^, mean±SD	23.33±2.89	24.53±3.14	<0.001	23.53±3.14	25.26±3.67	<0.001
FPG, mmol/L	5.68(5.33-6.16)	5.78(5.42-6.22)	0.002	5.68(5.36-6.12)	5.96(5.58-6.46)	<0.001
SCr, μmol/L	71(65-78)	80(72-89)	<0.001	54(49-60)	63(56-74)	<0.001
BNU, mmol/L	5.2(4.4-6.2)	5.5(4.7-6.4)	<0.001	5.1(4.3-6.1)	5.7(4.8-7.0)	<0.001
ALT, μ/L	18(14-25)	21(16-29)	<0.001	16(13-22)	18(14-27)	<0.001
AST, μ/L	22(19-26)	24(20-29)	<0.001	21(18-25)	23(19-29)	<0.001
Heart rate, bpm	75(67-83)	74(67-84)	0.478	78(71-85)	77(70-86)	0.940
TC, mmol/L	4.58(4.06-5.16)	4.75(4.18-5.39)	<0.001	4.80(4.26-5.40)	4.96(4.40-5.65)	<0.001
TG, mmol/L	0.94(0.70-1.36)	1.29(0.92-1.92)	<0.001	1.09(0.80-1.49)	1.51(1.06-2.06)	<0.001
HDL-C, mmol/L	1.43(1.19-1.73)	1.31(1.10-1.60)	<0.001	1.51(1.27-1.77)	1.36(1.14-1.59)	<0.001
LDL-C, mmol/L	2.49(2.08-2.94)	2.66(2.18-3.09)	<0.001	2.66(2.23-3.12)	2.82(2.35-3.33)	<0.001
Non-HDL-C, mmol/L	3.06(2.54-3.65)	3.38(2.80-3.97)	<0.001	3.24(2.73-3.85)	3.60(3.01-4.23)	<0.001

SBP: systolic blood pressure; DBP: diastolic blood pressure; BMI: body mass index; FPG: fasting plasma glucose; SCr: serum creatinine; BUN: blood urea nitrogen; ALT: alanine aminotransferase; AST: aspartate aminotransferase; TC: total cholesterol; TG: triglycerides; HDL-C: high density lipoprotein cholesterol; LDL-C: low density lipoprotein cholesterol; non-HDL-C: non-high density lipoprotein cholesterol.

### Correlation between blood lipids and SUA

A Spearman correlation analysis was used to estimate correlation between lipids and SUA in males and females. As shown in Table 2, in male group, SUA positively correlated to TC (r = 0.101, *P* < 0.001), TG (r = 0.272, *P* < 0.001), LDL-C (r = 0.107, *P* < 0.001), and non-HDL-C (r = 0.176, *P* < 0.001) and negatively correlated to HDL-C (r = -0.166, *P* < 0.001). The correlations between blood lipids and SUA were similar in female group. SUA also correlated to TC (r = 0.114, *P* < 0.001), TG (r = 0.322, *P* < 0.001), LDL-C (r = 0.144; *P* < 0.001), non-HDL-C (r = 0.220; *P* < 0.001), and HDL-C (r = -0.233, *P* < 0.001). The Spearman correlation coefficient between SUA and TG was the highest in both males and females. The Spearman correlation coefficient of non-HDL-C was higher than HDL-C in males and was higher than TC and LDL-C but followed HDL-C in females.

**Table 2:** Spearman correlations between lipid profiles and serum uric acid by genders

**Lipid indexes**	**Men**		**Women**	
	**Correlation coefficient**	***P*-value**	**Correlation coefficient**	***P*-value**
Total cholesterol	0.101	<0.001	0.114	<0.001
Triglyceride	0.272	<0.001	0.322	<0.001
HDL-C	-0.166	<0.001	-0.233	<0.001
LDL-C	0.107	<0.001	0.144	<0.001
Non-HDL-C	0.176	<0.001	0.220	<0.001

HDL-C: high density lipoprotein cholesterol; LDL-C: low density lipoprotein cholesterol; non-HDL-C: non-high density lipoprotein cholesterol.

### Association between blood lipids and hyperuricemia

As shown in Table 3, univariate logistic regression analysis showed that ORs for hyperuricemia were significantly associated with higher TC, TG, LDL-C, non-HDL-C and lower HDL-C (all *P* < 0.05) in male group. After adjustment for age, FPG, BMI, SBP, DBP, SCr, BUN, ALT, and AST, hyperuricemia was also associated with TC, TG, HDL-C, and non-HDL-C (all *P* < 0.05). We did not observe a significant association between hyperuricemia and LDL-C after multivariate adjustment. In female group, univariate logistic regression analysis showed that ORs for hyperuricemia were significantly associated with higher TC, TG, LDL-C and non-HDL-C and lower HDL-C (all *P* < 0.05). In multivariate models, hyperuricemia remained significantly associated with higher TG, non-HDL-C and lower HDL-C (all *P* < 0.05). We failed to observe significant associations of hyperuricemia with TC and LDL-C.

**Table 3:** Odds ratio and 95% confidence interval of hyperuricemia associated with blood lipid levels in both genders.

**Lipid profiles (mmol/L)**	**Odds ratio of hyperuricemia associated with higher lipid**			
	**Un-adjusted**	***P*-value**	Adjusted^*^	***P*-value**
Men				
Total cholesterol	1.29(1.18-1.41)	<0.001	1.17(1.06-1.29)	0.002
Triglyceride	1.41(1.31-1.52)	<0.001	1.35(1.25-1.46)	<0.001
HDL-C	0.54(0.44-0.66)	<0.001	0.70(0.55-0.89)	0.004
LDL-C	1.30(1.15-1.46)	<0.001	1.08(0.95-1.23)	0.257
Non-HDL-C	1.46(1.33-1.59)	<0.001	1.26(1.14-1.40)	<0.001
Women				
Total cholesterol	1.26(1.15-1.38)	<0.001	1.06(0.95-1.18)	0.271
Triglyceride	1.68(1.54-1.84)	<0.001	1.50(1.36-1.65)	<0.001
HDL-C	0.29(0.22-0.38)	<0.001	0.33(0.24-0.44)	<0.001
LDL-C	1.35(1.20 -1.53)	<0.001	1.11(0.96-1.27)	0.150
Non-HDL-C	1.53(1.39-1.68)	<0.001	1.26(1.13-1.41)	<0.001

^*^Covariates for adjustment including age, systolic blood pressure, diastolic blood pressure, body mass index, fasting plasma glucose, serum creatinine, blood urea nitrogen, alanine aminotransferase and aspartate aminotransferase. HDL-C: high density lipoprotein cholesterol; LDL-C: low density lipoprotein cholesterol; non-HDL-C: non-high density lipoprotein cholesterol.

### Comparison in AUCs between non-HDL-C and traditional lipid profiles related to hyperuricemia

**Figure 1:**
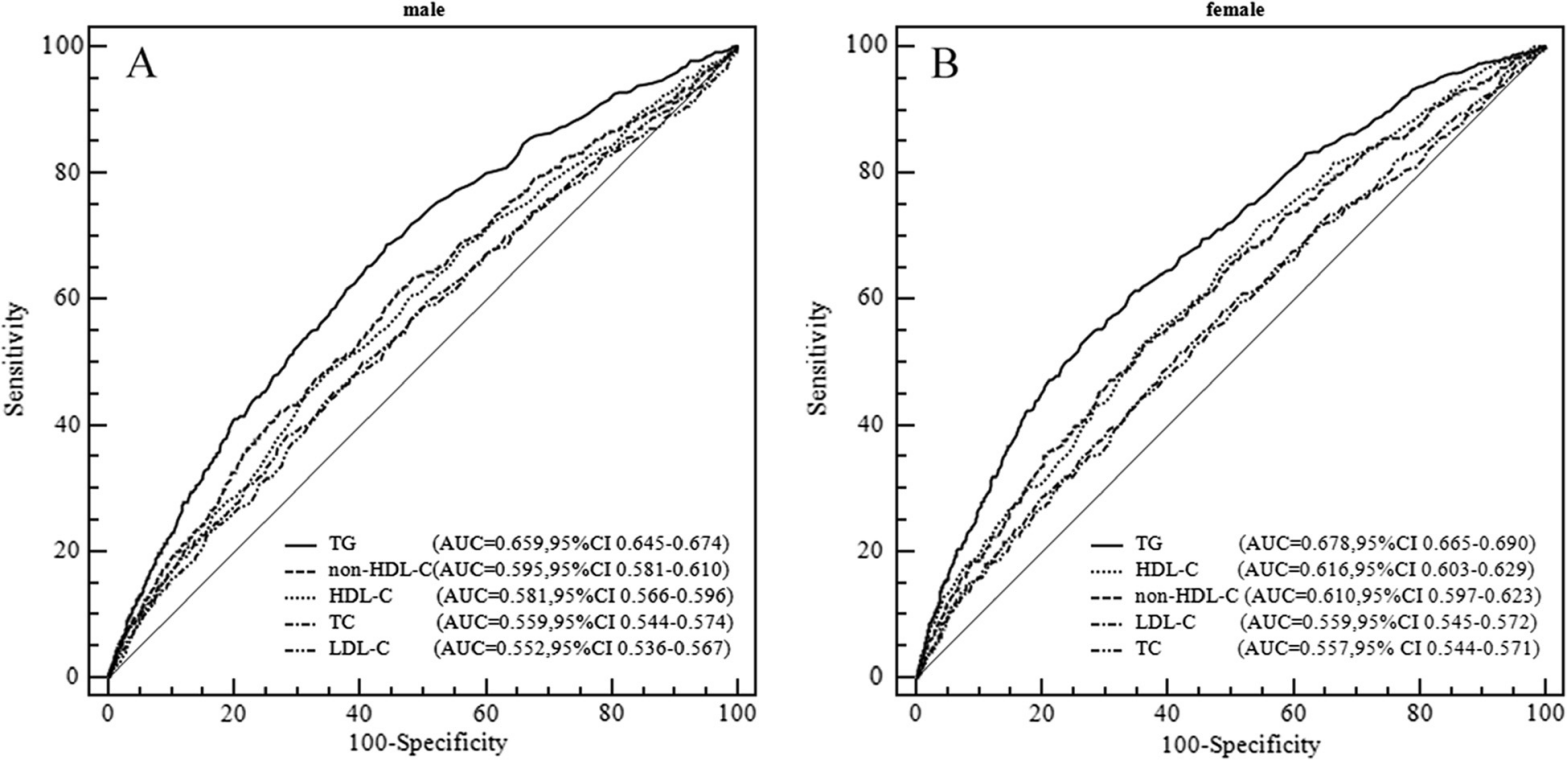
**Discriminatory powers of TG, TC, HDL-C, LDL-C and non-HDL-C for hyperuricemia by receiver operating characteristic (ROC) curves in males (A) and females (B).** AUC: areas under the curve; CI: confidence interval; TG: triglyceride; TC: total cholesterol; HDL-C: high density lipoprotein cholesterol; LDL-C: low density lipoprotein cholesterol; non-HDL-C: non-high density lipoprotein cholesterol.

Graphical comparisons of ROC curves of various blood lipids related to hyperuricemia in males (Figure A) and in females (Figure B) are presented in Figure 1. Figure A shows the discriminatory values of the five lipids related to hyperuricemia in men. AUC values for various lipids were as follows: 0.659 for TG, 0.595 for non-HDL-C, 0.581 for HDL-C, 0.559 for TC, and 0.552 for LDL-C. AUC was significantly greater for TG than other lipid profiles including non-HDL-C (all *P* < 0.001). Non-HDL-C was also found to have a larger AUC value than TC (*P* < 0.001) and LDL-C (*P* <0.001). There was no significant difference between non-HDL-C and HDL-C in AUC value (*P* = 0.333). There was no significant difference in the AUC value among HDL-C, TC and LDL-C. Figure B shows the discriminatory value of the five lipids related to hyperuricemia in women. The AUC values were 0.678, 0.616, 0.610, 0.559, and 0.557 for TG, HDL-C, non-HDL-C, LDL-C and TC, respectively. TG had the largest AUC value, and non-HDL-C had higher AUC value related to hyperuricemia than LDL-C or TC (all *P* < 0.001).

## Discussion

In this study, we found that TG was the best lipid index associated with hyperuricemia. Following TG, non-HDL-C was also significantly associated with hyperuricemia in both genders. Previous studies have examined the associations of lipid profiles such as TG, TC, LDL-C and HDL-C with hyperuricemia [13,14]. Recently, non-HDL-C was demonstrated to be more powerful in predicting CVD events [10] and highlighted as a key secondary target of therapy in patients with an increased TG [15,16], which implied that there may be a stronger association between non-HDL-C and CVD risks compared with other lipids. Some studies [17,18] have showed that non-HDL-C is associated with other risk factors of CVD including hypertension, diabetes, and overweight. However, no study has compared non-HDL-C with other lipid profiles in relation to hyperuricemia to date. Therefore, in addition to the relationship between non-HDL-C and hyperuricemia, we compared the discriminatory values of these lipid profiles in relation to hyperuricemia. We found that TG and Non-HDL-C had larger AUCs related to hyperuricemia compared with LDL-C and TC in males and females.

Our findings are consistent with other studies [19,20] that have reported a close link between serum TG and SUA in some groups. Conen and colleagues conducted a study in a population ongoing healthy examination, which demonstrated that TG was more strongly associated with SUA than HDL-C and TC [21]. Russo and colleagues reported that TG had a more closely relation to SUA than other lipids even in the healthy people [22]. The correlation coefficient between TG and SUA was higher than that between TC or LDL-C and SUA in the Verona Young men, even after adjustment for other potential covariates [23].

Non-HDL-C includes all lipoproteins that contain apolipoprotein B i.e. very low density lipoprotein, LDL-C, intermediate-density lipoprotein, chylomicrons and lipoprotein (a). Erdogan’s study showed that correlation coefficient of non-HDL-C to SUA (r = 0.382, *P* < 0.001) was slightly higher than that of TG (r = 0.379, *P* < 0.001) and obviously higher than those of TC, HDL-C and LDL-C in the healthy adults [24]. It is worthwhile to note that non-HDL-C can be accurately calculated based on all routine lipid profiles, fasting or non-fasting, at no further expense to the patient. Importantly, high non-HDL-C is treatable and has already been assimilated into current treatment guidelines.

Some previous studies found that treatment for hypertriglyceridemia with fenofibrate or atorvastatin reduced SUA levels [25,26]. In addition, an animal experiment study demonstrated that lowering uric acid with allopurinol prophylactically prevented fructose-induced hyperuricemia and hypertriglyceridemia [27]. All these studies indicated that TG and SUA might share a common metabolic mechanism so that some drugs can reduce TG as well as SUA levels. Keenan et al.’s study also suggested that increased uric acid via increased systematic inflammation predict insulin resistance, followed by dyslipidemia and hepatic steatosis [28]. In addition, some investigators tend to conclude that both hyperuricemia and dyslipidemia reflect the common lifestyles and dietary habits such as excessive alcohol consumption, high fat diet and physical inactivity [21,29]. Reduction of TG intake in diet has been shown to increase uric acid elimination. However, the effect was minimal and transient [30]. Thus, a combination of anti-hyperlipidemia and anti-hyperuricemia agents is a good option for the treatment of hyperuricemic patients with hypertriglyceridemia [31]. Some remedy drugs with dual effects of lowering both TG and SUA levels are needed to control the combined syndrome with hyperuricemia and hyperlipidemia.

This study has some strength that should be mentioned. This is a large-scale survey research in a middle-aged and elderly Chinese population and we firstly compared various blood lipid profiles including non-HDL-C in relation to hyperuricemia. Since men had a higher prevalence of SUA compared with women in current study, we studied the relations of blood lipids to hyperuricemia in men and women separately. The important co-variables such as hepatorenal indexes were also measured and evaluated in our study [32,33]. To some extent, these could control potential influence of some confounding factors including hepatorenal indexes on the relations of blood lipid profiles to hyperuricemia. However, the results of ROC analysis suggested that lipid indexes including TG and non-HDL-C were not good discriminate predictors for hyperuricemia in this study. Therefore, some additional factors besides lipid indexes should be considered in treating and preventing hyperuricemia, such as control of purine-rich foods, obesity or drinking [29].

There were several limitations in this study. First, this was a cross-sectional study and therefore a causal relationship between blood lipid profiles including non-HDL-C and risk of hyperuricemia could not be established. Secondly, data on other confounding factors such as diet, alcohol consumption and physical activity were not considered in this study, although the hepatorenal indexes can reflect accumulation of diet and alcohol consumption to some extent.

In summary, our results show that serum TG and non-HDL-C have stronger relations to hyperuricemia compared with other lipid indexes. Considering the increasing prevalence of hyperuricemia and its potential links with blood lipids, it is necessary to pay more attention to TG and non-HDL-C levels in these people with hyperuricemia.

## Competing interests

The authors declare that they have no competing interests.

## Authors’ contributions

JX, HP and QM carried out the studies. JX and HP performed the statistical analysis and drafted the manuscript. HP, QM, XZ, LH, JH and YZ conceived of the study, and participated in its design and coordination. YZ helped to draft the manuscript. All authors have read and approved the final manuscript.

## Author’s information

Juan Xu, Hao Peng and Qinghua Ma are co-first authors.
